# Compassionate Nursing Care for COVID-19 Patients in the ICU in the Western Region of Saudi Arabia: A Lived Experience Study

**DOI:** 10.7759/cureus.46954

**Published:** 2023-10-13

**Authors:** Tagwa Y Omer, Hawazen Rawas, Elham Bukhari

**Affiliations:** 1 College of Nursing, King Saud Bin Abdulaziz University for Health Sciences, Jeddah, SAU; 2 Nursing Education Department, King Abdulaziz Medical City, Jeddah, SAU

**Keywords:** compassionate nursing care, covid-19, saudi arabia, intensive care, lived experience

## Abstract

Introduction: The rapid spread and the severity of symptoms of COVID-19 led to an increasing number of critical cases that need to be admitted to intensive care units (ICUs) worldwide. Compassion is a principle of nursing practice and indicates the meaning of providing high-quality care in all units, especially in the ICU. It means that nurses know what is important to patients and when they should be there for them when it is needed. There is a paucity of literature from Saudi Arabia that explores how critical care nurses perceive compassionate care during COVID-19.

Objectives: The aim of this study was to investigate the lived experiences of critical care nurses providing compassionate care to COVID-19 patients at King Abdulaziz Medical City, Jeddah, Saudi Arabia.

Methods: A prospective, cross-sectional, descriptive phenomenological approach was utilized in this study. Data were collected from 13 ICU nurses through interviews. Collected data were transcribed and analyzed using Colaizzi’s data analysis method.

Results: Eleven out of the 13 ICU nurses hold a Bachelor of Science in Nursing (BSN) and have more than five years of experience. Despite all the challenges surrounding the care for critically ill COVID-19 patients, nurses provide compassion in different ways to show personal interest to the patients. Data analysis revealed five themes: (1) contemporary meaning and competencies for compassionate nursing care, including communication and the inability to freely use touch; (2) physical symptoms, including discomfort, body aches and headaches, and sleep disturbances; (3) emotional turmoil, where three sub-themes emerged, including fear and anxiety, uncertainty, and isolation and loneliness; (4) role changing, including frequent guidelines change, additional roles and responsibilities, and altruism; (5) professionalism, including cultural facets of care, teamwork, and support.

Discussion: The COVID-19 pandemic has caused major changes in nurses’ working environment and so their experience. The results of this study indicated that nurses working in the ICU experienced remarkable and massive physical, psychological, and emotional symptoms during the COVID-19 pandemic. The nurses extended the relationships to the patients’ families as they are at high risk of stress, anxiety, and depression. In addition, they were able to support them in dealing with the fear associated with the uncertainty of COVID-19. Also, results revealed that remote therapeutic relationships and psychotherapy can be credible and trustworthy alternatives to in-person care.

Conclusions: The results of this study indicated that ICU nurses provide compassionate care despite experiencing unprecedented and immense physical, psychological, and emotional symptoms during the COVID-19 pandemic.

Recommendations: Further study is recommended using other research methodologies. It is also recommended to conduct the same study in different cities for better generalization.

## Introduction

The aim of this study was to investigate the lived experiences of critical care nurses providing compassionate care to coronavirus disease 2019 (COVID-19) patients at King Abdulaziz Medical City, Jeddah, Saudi Arabia.

The COVID-19 outbreak has been declared a global pandemic by the World Health Organization (WHO) that is rapidly changing and is affecting communities from all over the world [1]. Approximately 15% of reported cases globally developed severe complications with a case fatality rate of 4.2% [2]. Most of the critical cases developed respiratory failure, septic shock, and/or multiple organ dysfunction [3]. It is estimated that around 5-10% of severe cases require intensive care [4].

The rapid spread of COVID-19 and the severity of symptoms led to an increasing number of critical cases that need to be admitted to intensive care units (ICUs) worldwide. This makes the healthcare workers (HCWs), particularly those working in ICUs, face a lot of challenges, including anxiety and stress due to a lack of adequate personal protective equipment, staff shortages, and a shortage of beds and mechanical ventilators [4]. Furthermore, they worried about several issues, including the potential risk of exposure, the uncertainty of the organizational support if they develop an infection, and a rapidly changing practice environment that differs from what they are familiar with [5]. Research evidence indicates that health professionals may experience various psychological problems when working in high-pressure or risky situations, such as during the time of the pandemic [4,6].

HCWs witnessing the patients' prolonged suffering and death of patients with COVID-19 is associated with post-traumatic stress symptoms and their perceived inability to alleviate the suffering of those in their care [6]. As a result, critical care nurses are at heightened risk of having severe emotional stress, which negatively impacts safety outcomes, negative patient experience, and compassionate care [7,8].

Compassionate care is a concept that indicates the meaning of providing high-quality care [9]. It is defined as knowing what is important to patients and when nurses should be there for them when it is needed. Compassionate care is an essential principle of patient-centered care, where the nurses show their compassionate care toward the patients through their relationships with them, which should be based on empathy, respect for patients’ opinions, values and beliefs, and dignity [9,10].

It has been reported that patients feel compassionate when nurses build a connection with them and give them their attention. Moreover, the awareness of the patient's situation during hospitalization, such as the experiences of emotional issues, family issues, or the lack of independence, was also viewed as being compassionate [11]. Seven dimensions of compassion were identified [9]: “attentiveness, active listening, the naming of suffering, involvement, helping, being present, and understanding.” Therefore, compassionate care is a fundamental aspect of the nursing profession and staff nurses must demonstrate compassion in their practice.

Providing compassionate care not only benefits the patient, but it also benefits the nurse. Empirical research has demonstrated that compassionate care improves patients’ outcomes and the quality of patients’ care, increases coping abilities, and empowers patients. Further, compassion is associated with beneficial effects for nurses, including greater job satisfaction, improved engagement of patient-nurse relationships, and reduced burnout [12]. Compassionate care also benefits healthcare organizations by promoting the standards of care and improving patients’ satisfaction regarding the provided care [13].

Despite all this, literature has discussed compassion as the missing practice among nurses. According to recent evidence, only 53% of the hospitalized patients perceive care to be compassionate [9]. Moreover, according to the Agency for Healthcare Research and Quality (2013), 10.8% of patients believed that the healthcare providers do not build good relationships with them during the hospitalization as they do not spend time talking or listening to them, discussing their procedures or any issues related to that procedure [10].

Empirical evidence has demonstrated that some factors related to the workplace or staff could be barriers to compassion in practice. Such barriers at the organizational level include the inconsistency between the workload and its allocated time, unawareness of the needs of the nurse, and the lack of role modeling for compassionate care. Additionally, the nurses’ personal and professional attitudes in achieving compassionate care could be barriers at the individual level [10].

Although nurses and patients believe that compassion is one of the most important professional values, there is a paucity of Saudi literature that explores how critical care nurses perceive compassion during COVID-19.

## Materials and methods

Design

The purpose of this study is to gain insight into the personal lived experiences of nurses caring for patients with COVID-19 in the ICU. A descriptive phenomenological inquiry design was used to carry out this study. Phenomenological inquiry is a method used to explore and describe the lived experience of individuals through prolonged engagement with participants, which enables the emergence of patterns and relationships [14]. Phenomenology provides deeper and more meaningful understanding and can be considered as a source of evidence beyond existing understanding [15]. In this study, phenomenology is used to emphasize the investigation of how individuals make meaning of their experiences [16]. Descriptive phenomenology founded by Edmund Husserl (1859-1938) aimed to identify the meaning of an individual’s lived experiences or to extract meaning from their everyday life [17].

Ethical considerations

Institutional review board approval was secured from King Abdullah International Medical Research Center (KAIMRC) before data collection started.

All participants were asked to sign an informed consent that was explained by the researcher and contains the objectives of the study, ensuring that participation is voluntary and that participants can withdraw from the study at any time. Participants were provided with a copy of the consent for the record.

Collected data were kept in a safe place. The recorded interviews and transcripts were kept on the researcher's laptop with a password, which no one other than the researchers could have access to. Following KAIMRC policy, the data will be disposed of after three years from publication, deleting all data from the laptop. Confidentiality and anonymity were maintained throughout all the stages of the research, and no names or identification of participants were mentioned or written.

Participants

Data were collected from nurses working at King Abdulaziz Medical City, Ministry of the National Guard, Jeddah, in the Western Region of Saudi Arabia. The hospital has 45 ICU beds. The hospital, like all other hospitals, received patients who suffered from COVID-19 during the pandemic from January 2020 till the time of data collection in January 2022.

Purposeful, snowball sampling was employed for this study. According to Palinkas et al. [18] and Creswell [19], it is appropriate for a researcher to select the sample based on the knowledge of the population, its elements, and the nature of the research aims. The researchers chose to use a purposeful, snowball sampling method because of the need to know the culture, the types of patients, and the nature of the disease. Snowball is an intentional sampling strategy that identifies participants who know other potential participants who fulfill the inclusion criteria and have knowledge about the phenomenon under study. Once participants are identified, they may refer new participants to the study [18,20].

Inclusion criteria

Nurses with a minimum of one year of employment at the hospital, who are currently assigned to the ICU for COVID-19 patients, and are willing to voluntarily participate in the study were included.

Data collection

Unstructured, open-ended interviews were used to collect data for this study. It is the most popular method used in phenomenological research [21]. Due to the nature of COVID-19 and the isolation process, the interviews were conducted through Zoom video calls (Zoom Video Communications, San Jose, CA). All interviews were audiotaped with the date and time specified. The recorded audio tapes were then transcribed.

Verbatim transcripts were organized and prepared for analysis. Data collection continued till saturation was reached. Saturation is the point when no new information or concepts are observed in the data or when data redundancy is achieved [22].

Data analysis

Collected data were analyzed using Colaizzi's method [23], as explained by De Chesnay [24]. Colaizzi’s data analysis method involves seven steps: (1) reading and rereading the participants’ descriptions of the phenomenon to acquire a feeling for their experience and to make general sense of their experience. (2) Extracting significant statements that pertain directly to the phenomenon under study. (3) Formulating meanings for these significant statements to illuminate hidden meanings. (4) Categorizing the meanings into clusters of themes and confirming consistency between the emerging findings and the participants’ stories without giving in to the temptation to ignore data that do not “fit.” (5) Integrating the findings into an exhaustive description of the phenomenon under study; describing includes coding segments of text for topics, comparing topics for consistent themes, and bridging themes for their conceptual meanings, which leads to creating a prototype of a theoretical model about the phenomenon studied. (6) Validating the findings by returning to the study participants to ask how the universal description compares with their personal experiences. An example of the question asked was to elaborate on the ethical consideration of providing compassionate care to patients with COVID-19. (7) Incorporating any changes offered by the participants into the final description of the phenomenon.

Academic rigor

Consolidated criteria for reporting qualitative research were used in this study. For the trustworthiness of data, the authors focus on credibility, transferability, dependability, and confirmability [24,25]. To address credibility, Lincoln et al. [25] identified several steps to address this point. First, researchers had prolonged engagement with participants to learn the culture and build trust. Second, researchers made two transcriptions for each interview by listening to the audio recording on two separate occasions. Then researchers merged the two transcriptions into one final version. Each final transcription was read line by line two to three times to identify the main themes [25]. This procedure provided a richer, more meaningful, and more credible data set. Moreover, the credibility of the current study during data analysis and interpretation was enhanced by showing apparent contradictions in data. Transferability was achieved by selecting a purposeful sample from different ICUs to illuminate the phenomena being studied. In addition, a thick description of the contextual background of the research setting and the participants and credible interpretation are necessary. The dependability of research data is satisfied by using the interview guide to be consistent. In addition, the transcription strategy was universally similar for all interviews conducted. Digitally recorded interviews were added as another way of enhancing the dependability of obtained data by minimizing any systematic bias and producing plausibility of the account made by interviewees.

## Results

Demographic characteristics of the participants

Thirteen ICU nurses participated in this study, including four male and nine female nurses, aged 26-49 years. Table 1 presents the demographic characteristics of the participants.

**Table 1:** Demographic characteristics of the participants

Characteristics	No.	Percentage	Mean
Total number of participants	13		
Age			32.5 years
Sex			
Male	4	30.8%	
Female	9	69.2%	
Marital status			
Married	6	46.1	
Single	5	38.5%	
Divorced/widowed	2	15.4%	
Nationality			
Saudi	5	38.5%	
Expatriate	8	61.5%	
Educational level			
Diploma	2	15.4%	
Bachelor of Science in Nursing	11	84.6%	
Years of experience			
<5 years	4	30.8%	
5-10 years	5	38.4	
>10 years	4	30.8%	

Themes

Five themes emerged from the data collected from the participants. Each theme includes several sub-themes that are related to it. Participants expressed their understanding, meaning, and perception of the compassionate care they provided to their patients who have COVID-19 in the ICU.

The delivery of compassionate care to patients with COVID-19 in the ICU is affected by many factors and circumstances. The result of this study presents an in-depth understanding of the meaning and lived experience of nurses taking care of patients with COVID-19 in the ICU. The five themes that emerged are (1) contemporary meaning and competencies for compassionate nursing care, (2) physical symptoms, (3) emotional turmoil, (4) role changing, and (5) professionalism. Each theme includes several sub-themes that are related to it. Table 2 presents the five themes and sub-themes.

**Table 2:** Emerged themes and sub-themes

	Theme	Sub-themes
1	Contemporary meaning and competencies for compassionate nursing care	Therapeutic communication and inability to freely use touch
2	Physical symptoms as a sign of compassion fatigue	Discomfort, body aches and headache, and sleep disturbances
3	Emotional turmoil as a sign of compassion fatigue	Fear and anxiety, uncertainty, and isolation and loneliness
4	Role changing	Frequent guidelines change, additional roles and responsibilities, and altruism
5	Professionalism	Cultural facets of care, teamwork, and support

Theme 1: contemporary meaning of compassionate care

Nurses participating in this study indicated that they have a new meaning of compassionate nursing care. Despite all the challenges surrounding the care for critically ill COVID-19 patients, nurses provide compassion in different ways to show personal interest to the patients.

Therapeutic Communication

The pandemic environment created fear and anxiety in nurses. Building therapeutic relationships in this environment is very challenging, but nurses indicated that they still provide care based on therapeutic relationships as a core of nursing skills. The nurses indicated that they build relationships with families of patients through telephone calls where a phone line was established for open communication with patient’s families.

N4: “Although PPEs are essential to prevent the spread of COVID-19, but they are considered as barriers to build therapeutic relationship. We still show patients and family our empathy and respect. We honestly care for them as human beings.”

N8: “We call and receive calls from families and if the patient is wake, we arrange video calls with families. We listen to their concerns, and we are being consistent with them. We accept them genuinely.”

N1: “Even if patient is unresponsive, we try our best to communicate with families to let them know that we are around and taking care of their loved ones.”

N6: “We continuously communicate with patient’s families to let them know the patient’s status. We have a phone extension assigned to answer the family calls regarding their patients and if the patient is awake and conscious, we help them to make Video calls with their family.”

N3: “I support and encourage family members to seek help if needed, especially having a patient with COVID-19 very stressful and emotionally draining. We communicate and make referrals to social workers if needed.”

N13: “I teach family the importance of self-care and recommend to them to have the resources needed as social workers and stress-reducing activities.”

Nurses also indicated that they use facial expressions to reassure patients and encourage them.

N11: “Due to limited direct contact with patients as indicated in the practice guidelines, we take any chance to be near the patient to use facial expression as communication to convey and demonstrate our support and compassion.”

N9: “I tried any chance of entering my patient’s room to reassure him by smiling at him or nodding to him to make him feel comfortable.”

Inability to Freely Use Touch

Due to COVID-19 and the use of personal protective equipment (PPE), nurses indicated that they cannot directly touch the patients, but still provide compassionate care.

N6: “We are trying our best to convey our compassion and our empathy to patients without touching them, we let them feel we are beside their beds.”

N1: “Using PPE prevents us from touching our patient, but they feel how we are very compassionate in our practice.”

Theme 2: physical symptoms as a sign of compassion fatigue

The concept of compassion fatigue is a consequence of exhaustion caused by witnessing the suffering of patients, stressful work conditions, and inadequate utilization of measures to promote self-care. Compassion fatigue has physical, psychological, and emotional symptoms. These symptoms could be barriers to providing compassionate care to patients with COVID-19 in the ICU.

Discomfort, Body Ache, and Headache

Nurses expressed that they experienced physical symptoms for longer than usual since the start of the COVID-19 pandemic.

N12: “The long shift hours and increased workload are the factors that lead us to have symptoms. I have a continuous headache and body ache. I am diagnosed now with migraine headache, and I am on treatment.”

N9: “Every day I go home after the shift-day or night- complaining of body ache and headache. Wearing PPE for long time causes us to feel sick, all have physical symptoms.”

Sleep Disturbances

Participants identified the experience of taking care of patients with COVID-19 makes them very exhausted and have symptoms of physical illnesses.

N3: “I go home after my shift very tired but still cannot sleep well, sometimes I keep thinking about work which keeps my mind and making it difficult to sleep.”

N4: “All the time while sleeping, I am hallucinating because of the inadequate time to rest. We are supposed to feel tired after shift and sleep deeply but unfortunately, we don’t.”

Theme 3: emotional turmoil as a sign of compassion fatigue

Fears and Anxiety

Participants expressed feelings of fear and anxiety. The participants became very emotional during the interview while they were expressing their feelings.

N2: “We all have great fear especially when we have the few first cases. We have fear of the infection and fear of transmitting the infection to our families, the level of anxiety is very high between us.”

N13: “I’m scared and worried about myself and my family. At the beginning, we don’t know enough information needed. I was very anxious; we all tell each other how we are afraid.”

N12: “COVID-19 induced a lot of fears in our lives. The information we receive every day was from different sources and not similar, every day new guidelines. You know what? Even the WHO is changing their news every day. We are afraid. I stop watching TV.”

Uncertainty

The majority of participants expressed a feeling of uncertainty due to the lack of enough informal needs.

N4: “We are not certain what to expect regarding the Pandemic. Although we follow the guidelines sent to us from the Infection Control Department at our hospital, we still receive information and news through the media. Social media is very conflicting and sends abundant amount of confusing information.”

N5: “I am uncertain about my status, I always on the way home from my shift think of how to protect my family. Because of the different manifestations of COVID-19, we become uncertain on how to behave with our families.”

N10: “The infection control department is updating us every day. I am not sure of what is next. Changes coming everyday.”

Isolation and Loneliness

Participants reported that due to the quarantine and isolation in the unit, they feel loneliness and separated from the world.

N5: “Other colleagues and society look at me as if I have COVID-19 infection. People try to get away from me because they are scared that I will carry the infection. During this pandemic, I feel we are isolated and there is stigma on nurses.”

N7: “I feel rejected by others. When I am socializing outside work, I do not tell people that I work with patients who have COVID-19. I did not tell my mother that I work with infected patients because she will be scared.”

N2: “The stigma and social isolation due to COVID-19, let all nurses feel loneliness. We as nurses, we stay longer time at the bedside than any other health professionals. Some people distance themselves from us and they think we can transmit the disease to them.”

Theme 4: role changing

During COVID-19, nurses work in highly complex and uncertain environments, where all individual resources have to be mobilized to quickly adapt to the numerous changes imposed by the pandemic.

Frequent Guideline Changes

During the COVID-19 pandemic, nurses performed tasks more than their regular job description.

N2: “We usually receive instructions and directions from the ICD and the Nursing Administration, but the guidelines change very frequently. This cause confusion and affects the work.”

N11: “We become very confused because of the different sources of information. The WHO Guidelines changes daily.”

N9: “It’s very confusing when guidelines were changing frequently. The sources of information are contradictory sometimes. That affects our work practices.”

Additional Roles and Responsibilities

During the pandemic, most of the supporting staff in the hospital were working from home, so nurses were performing tasks that they were not used to taking.

N5: “Our roles went beyond the ICU. We are involved more in public health with infection prevention, health education and health promotion in the community.”

N8: “As a nurse, we are now more involved in planning to maintain efficient use of supplies and materials at the level of our unit and all the hospital.”

N7: “Usually ICU is a very closed work environment, but during the Pandemic, our responsibility became out of the ICU. We are engaged in public awareness regarding the disease and the Pandemic nature and disseminating facts and correcting myths.”

Altruism

Altruism is one of the professional values of nursing care where nurses focus on the welfare of their patients and are willing to make sacrifices for the needs of patients and their families.

N5: “We work continuously even without taking breaks because patients need us. We put our patient’s need before ours. We also consider the needs of our colleagues.”

N6: “I don’t see my family sometimes for a whole week because we were in quarantine in the hospital housing assigned for us.”

N10: “We are relocated inside the hospital housing during the quarantine, so we didn’t meet our families. But we support each other.”

N7: “Despite the hard work we do, we feel helpless. We are trying our best to help the patients and patient’s families. We use our own cellphones to let patients communicate with their families.”

Theme 5: professionalism

Several factors during the COVID-19 outbreak have affected the nurses' working conditions, especially the nurses' professional commitment to the health profession including nursing.

Cultural Facets of Caring

During the COVID-19 pandemic, nurses practice based on their beliefs, attitudes, and norms explanations. These cultural elements are considered to play a key role in the adoption of prevention and management of the virus.

N1: “I recognize the importance of religious aspect for the Muslim patients, and I tried to learn some Arabic language words to make patients feel comfortable.”

N9: “Although I am not Muslim, I help patients to pray in their beds and allow them to play Quran if they wished to do so.”

N12: “Due to the language difference, I always ask Arabic employ to help interpret to ensure that I understand what patients say and make sure he understands me.”

Teamwork and Support

The widespread use of COVID-19 impacts the work dynamic in healthcare institutions and has demanded quicker learning about teamwork.

N2: “I feel we became united during this difficult time; I never have this feeling of a family in my life, my colleagues in the ICU and our administration become one team, have one goal and one voice.”

N5: “The sense of belonging I have is great. Though it’s a very difficult time, but not thinking about himself only. We think as one Army and that is the ICU team.”

N10: “We support each other, and we are obligated to the team. We take care of each other while taking care of our patients.”

N13: “When we feel one of us is anxious or stressed, we all run to help and support. We really became one family during this Pandemic. We even take care of each other kids because of quarantine no sitters can come to the house. We buy groceries for each other and even cover shifts of each other if needed.”

## Discussion

The HCWs responded to the COVID-19 pandemic with valuable efforts to address physical, emotional, social, and spiritual needs. In addition, they were able to develop relationships with the patients and their families and support them in dealing with stress and fear [26]. For example, despite the challenges of the COVID-19 pandemic, nurses continued building a nurse-patient relationship through therapeutic communication. In addition, this global outbreak had a great impact on communication and relationships with patients and their families given the need to maintain isolation and social distance [27]. The nurses in this study extended the relationships to the patients’ families, as the relatives of critically ill patients are at high risk of stress, anxiety, and depression [28,29]. To relieve these symptoms, participants were instructed to call and receive calls from family members of their patients. Results of a study investigating the perceived changes to the psychotherapeutic relationship during the pandemic revealed that remote therapeutic relationships and psychotherapy can be credible and trustworthy alternatives to in-person care [30].

Nurses in the ICU found difficulty communicating with ventilated patients. Communication challenges with patients were aroused due to PPE and fear of infection. Nurses indicated that they are communicating with patients on ventilators by non-verbal techniques such as body gestures or facial expressions. A study revealed that nurses could not communicate with the patient as they should in a patient-caregiver relationship due to the protective gear used by nurses and the medical condition of the patient, but nurses used other gestures to aid communication, such as body language and other writing methods [31].

Touch is an essential and important form of nonverbal communication, which is considered central to the provision of comfort and an intentional way to convey affection, reassurance, and compassionate care in times of distress [11,32]. The nature of COVID-19 as a contagious infection mandates that health workers limit their direct contact with patients. Despite these, participants conveyed their compassionate care to their patients. Patients with COVID-19 conveyed that they are deeply cared for through the physical presence of health workers [33].

Family support includes education, involvement in care, and support in decision-making. The care of patients with COVID-19 extended beyond reaching family members. Restricted visits and limited access to patients’ rooms separate and isolate the patient from their families and friends. Nurses were addressing the physical and emotional needs of the patients as well as their families [34]. The family support team was very variable as emotional support through providing information about the patient's condition by calling or receiving calls from their family members. In addition, the families expressed their need for extended support even after the death of their patients [35]. Additionally, the admission of family members to the ICU causes anxiety to the entire family [36]. In the case of coronavirus, the anxiety level increases in such families. Therefore, decreasing or controlling the patient and family’s anxiety is an important role of the nurses to speed up the patient’s recovery [37]. Using a liaison nurse to communicate with patients and their families could reduce the development of high levels of anxiety [38].

Studies revealed that infectious disease outbreaks have psychological and physical symptoms among HCWs [39,40]. According to Namikawa et al. [41], HCWs who are engaged with critically ill COVID-19 patients have a 25% frequency of experiencing physical symptoms. Specifically, this frequency is higher among nurses with a percentage of 39.5% (p < 0.01). Several studies indicated that HCWs reported physical symptoms commonly such as headache, muscle tension, body ache, and fatigue [39].

Moreover, during the previous outbreak of severe acute respiratory syndrome (SARS) and Middle East respiratory syndrome (MERS), studies revealed the prevalence of sleep disturbances among HCWs [42]. In 2021, a meta-analysis conducted regarding the prevalence of insomnia among HCWs found that the prevalence of insomnia among HCWs during the COVID-19 pandemic was 36.36% (p = 0.006) [43]. Similar results were found in other studies regarding sleep disturbances of HCWs during various pandemics [44-46].

At the time of the COVID-19 pandemic, nurses as frontline HCWs faced great risks of mental and psychological health issues. Studies have found that nurses had moderate to high levels of fear [47]. In another study conducted in Saudi Arabia, results revealed that 10.7% of nurses had mild, 73.5% had moderate, and 15.5% had severe degrees of fear and anxiety [48]. This result is supported by the study conducted in Jordan, which reported elevated levels of fear among health workers [49].

Due to the quick spread of the virus and various sources of information, the participants expressed their feelings of uncertainty, especially during the early stage of the pandemic. A phenomenological study found that one of the major themes that emerged was uncertainty [50], where participants explained uncertainty that may be a result of lack or delayed communication of leadership. In addition, uncertainty arises when the need of the pandemic is not aligned with the needs of the patient and also can be caused by the disconnection between policies and frontline practice related to the COVID-19 decision [50]. According to the study by Moi et al. [51], nurses experienced uncertainty due to the lack of information and clear guidance, especially at the beginning of the pandemic.

The nature of the COVID-19 pandemic brought lockdown and social distancing in the community. All nurses who participated in the study mentioned social isolation as an issue [52]. One of the themes in a study [34], was managing isolation, fear, and increased anxiety, in which the participants expressed their fear of contracting and spreading the COVID-19 virus. Therefore, this will make people live in isolation and feel lonely. These results were incongruent with Razu et al. [53]. Separation from the family and living in the hospital accommodations was intolerable [54].

Frequent changes in the guidelines regarding COVID-19 prevention and management affect the delivery of care and the nature of the practice of healthcare professionals; therefore, leading to changes in the policies and practices [34]. Nurses working during the COVID-19 pandemic revealed that constant changes in the protocols and guidelines affect their clinical performance and the way they deal with their patients [55].

The pandemic required nurses to gain complex competencies to take care of patients [34]. To respond to the contagious nature of COVID-19 in the complex healthcare environment, nurses’ roles were extended to include testing, triage, and management as well as their role in prevention in the community [56]. In addition, to direct patient care during the pandemic, nurses are performing other tasks such as filling in for other HCWs as a result of the quarantine and the limited number of HCWs entering patient’s rooms [34]. Participants of the study viewed their experience as a new opportunity to new strategies and protocols for their professional life [54].

Altruism is part of the moral practice of nursing, and of the professional values that represent the standard of nursing care [57]. Results of a study conducted on the lived experience of altruism and sacrifices of nurses during the COVID-19 pandemic indicated that the nurses had moral obligations to respond to the patient's needs and the healthcare system's needs [58]. Nurses’ willingness and dedication to serve the ill patient during the pandemic was clearly increasing despite the fear and anxiety [58]. Iranian nurses expressed that although they are self-sacrificing while providing nursing care to patients with COVID-19, they experienced professional growth and transcendence [59,60].

In the nursing field, professional and moral values guide and shape clinical practice professionally and ethically [61]. Nurses promote their clinical practice according to their professional ethical values [62,63]. Nurses were found to have a sense of professional obligations [54].

Providing culturally competent care is an important professional value to nursing practice. One of the lessons learned during COVID-19 was the importance of considering culture and culturally competent care, which can contend the COVID-19 outbreak in high-risk communities [64]. Health professional workers dealt with several issues related to culture that may play a role in the prevention and management of COVID-19, such as the method of greeting and handshaking, and congregation praying [65]. Nurses are distributing the Sharia Council Fatwa ruling laws to educate people about the risks and prevention of COVID-19 [66]. During the lockdown, a higher level of religiosity was associated with a lower level of stress regardless of the type of religious practice [66].

The response to the COVID-19 pandemic requires a redesign of services to have enhanced collaboration, engagement, creativity, innovation, and knowledge sharing between the teams and greater collaboration is one of the important aspects of changes during the COVID-19 pandemic [67]. A systematic review in primary health care revealed the importance of teamwork support [68]. Collaboration and cooperation among nurses and other healthcare teams contribute to the management of middle and complex COVID-19 cases [69].

Limitations of the study

The study had a limited sample size. The recruitment process and the volunteering participation in this study are considered as limitations. This research was only conducted in the western region of Saudi Arabia, which may affect the generalization of the results. Due to the nature of COVID-19 and the isolation process, the interviews were conducted through ZOOM video calls, which may limit capturing some of the elements from participants.

Recommendations

Further study is recommended using other research methodologies. We also recommended conducting the same study in different cities for better generalization.

## Conclusions

The COVID-19 pandemic has caused major changes in nurses’ working environment and their experience. The results of this study indicated that ICU nurses experienced unprecedented and immense physical, psychological, and emotional symptoms during the COVID-19 pandemic. Understanding these experiences provides insights into areas that must be addressed to build and sustain an ICU nurse workforce. Studies are needed to further describe nurses’ experiences during the COVID-19 pandemic and identify effective resources that support ICU nurses' well-being. To improve the compassionate care by nurses, the organizations’ leaders should support them by considering human resources, workload, and the required skills.

This study's results can help policymakers and nurse leaders to better understand the factors affecting the experience of nurses taking care of patients with COVID-19 in the ICU and actively engage in supporting nurses both during and following the pandemic.
